# Lower Conditioning Leisure-Time Physical Activity in Young Adults Born Preterm at Very Low Birth Weight

**DOI:** 10.1371/journal.pone.0032430

**Published:** 2012-02-27

**Authors:** Nina Kaseva, Karoliina Wehkalampi, Sonja Strang-Karlsson, Minna Salonen, Anu-Katriina Pesonen, Katri Räikkönen, Tuija Tammelin, Petteri Hovi, Jari Lahti, Kati Heinonen, Anna-Liisa Järvenpää, Sture Andersson, Johan G. Eriksson, Eero Kajantie

**Affiliations:** 1 Department of Chronic Disease and Diabetes Prevention, National Institute for Health and Welfare, Helsinki, Finland; 2 Children's Hospital, Helsinki University Central Hospital, Helsinki, Finland; 3 Institute of Behavioural Sciences, University of Helsinki, Helsinki, Finland; 4 LIKES Research Center for Sport and Health Sciences, Jyväskylä, Finland; 5 Department of General Practice and Primary Health Care, University of Helsinki, Helsinki, Finland; 6 Unit of General Practice, Helsinki University Central Hospital, Helsinki, Finland; 7 Vasa Central Hospital, Vasa, Finland; 8 Folkhälsan Research Centre, Helsinki, Finland; University of Cape Town, South Africa

## Abstract

**Background:**

Adults born preterm at very low birth weight (VLBW, <1500 g) have elevated levels of risk factors for cardiovascular diseases and type 2 diabetes. Preliminary observations suggest that this could partly be explained by lower rates of physical activity. The aim of this study was to assess physical activity in healthy young adults born preterm at very low birth weight compared with term-born controls.

**Methodology/Principal Findings:**

We studied 94 unimpaired young adults, aged 21–29 years, born at VLBW and 101 age-, sex-, and birth hospital-matched term-born controls from one regional center in Southern Finland. The participants completed a validated 30-item 12-month physical activity questionnaire and the NEO-Personality Inventory based on the Big Five taxonomy, the most commonly used classification of personality traits. Yearly frequency, total time, total volume and energy expenditure of conditioning and non-conditioning leisure-time physical activity (LTPA) and commuting physical activity were compared between VLBW and term-born subjects. A subset of participants underwent dual-energy x-ray absorptiometry for body composition measurement. Data were analyzed by multiple linear regression. Compared with controls, VLBW participants had lower frequency [−38.5% (95% CI; −58.9, −7.7)], total time [−47.4% (95% CI; −71.2, −4.1)], total volume [−44.3% (95% CI; −65.8, −9.2)] and energy expenditure [−55.9% (95% CI; −78.6, −9.4)] of conditioning LTPA when adjusted for age, sex, body mass index, smoking, parental education and personality traits. Adjusting for lean body mass instead of body mass index attenuated the difference. There were no differences in non-conditioning LTPA or commuting physical activity.

**Conclusions/Significance:**

Compared with term-born controls, unimpaired VLBW adults undertake less frequent LTPA with lower total time and volume of exercise resulting in lower energy expenditure. Differences in personality that exist between the VLBW and term-born groups do not seem to explain this association.

## Introduction

People born preterm at very low birth weight (VLBW, <1500 g) have higher levels of risk factors for chronic non-communicable diseases than their peers born at term. These risk factors include impaired glucose regulation [1], [2], higher blood pressure [3]–[8], lower bone mineral density [9] and diminished lung function [10]. Evidence for increased risk of disease outcomes in people born preterm exists at least for type 2 diabetes [11]–[13], stroke [14] and osteoporosis [9]. Type 2 diabetes, high blood pressure and osteoporosis are all strongly related to lifestyle; being physically active and fit reduces the risk of developing these conditions [15]–[18]. Regular strenuous physical activity and maintenance of cardio-respiratory fitness reduces cardiovascular disease risk factors particularly in subjects born at low birth weight [19].

Previous studies have suggested that adolescents or young adults born severely preterm perceive their physical abilities as poorer [20], [21], participate less in sports [21], [22] and undertake less leisure-time physical activity (LTPA) [22], [23] than their term-born peers. However, physical activity assessment in all these studies was based on only a small number of questionnaire items and more specific information is needed for planning preventive measures and guiding exercise habits in the most optimal direction.

VLBW subjects show different personality traits compared with term-born peers; they are more conscientious, agreeable and show less neuroticism [24]. Of personality traits, conscientiousness and extraversion have previously been linked with higher, and neuroticism with lower levels of physical activity [25]. It is unknown to what extent personality traits contribute to physical activity among VLBW subjects.

Our primary aim was to examine the effect of being born preterm at VLBW on physical activity, specifically on conditioning and high intensity physical activity. We compared the frequency, total time, total volume and energy expenditure (EE) of conditioning LTPA, non-conditioning LTPA, commuting physical activity and vigorous physical activity between subjects born at VLBW and term-born controls. We used a detailed questionnaire specifically designed for this purpose. In addition, we examined whether the relation between prematurity and physical activity is dependent on personality traits.

## Methods

### Participants

The participants come from the Helsinki Study of Very Low Birth Weight Adults, a follow-up cohort of all subjects born preterm at VLBW between 1978 and 1985 and discharged alive from the neonatal intensive care unit of Children's Hospital at Helsinki University Central Hospital, the only tertiary neonatal centre in the Uusimaa province of Finland. The control subjects were selected from the same birth hospital; based on hospital data the following available term-born singleton matched for sex and appropriate for gestational age was included in the control group. The original cohort consists of 335 VLBW subjects and 373 controls of whom 255 and 314 were living in the greater Helsinki area at the time of the first clinical examination and were thus invited to the study. Of the subjects invited, 166 VLBW and 172 controls attended a detailed clinical examination during 2004–2005 [1], [9]. The data we now report on are based on a follow-up study carried out during 2007–2008 [26], [27]. For this follow-up we invited the participants of the first visit, except for 25 individuals who were not invited because of developmental delay (n = 1), earlier refusal for future contact (n = 4), being abroad (n = 11), being untraced (n = 2) and being ineligible for glucose metabolism studies which were included in the follow-up visit (n = 7; pregnancy, medication, type 1 diabetes). We thus invited 159 VLBW and 154 control subjects of whom 113 (71.1%) and 105 (68.2%) participated. Of these, 12 and 3 subjects did not complete the physical activity questionnaire. Of the 101 VLBW and 102 control subjects with physical activity data available, we further excluded 7 and 1 subjects due to cerebral palsy, developmental delay, blindness, hearing deficit or other condition potentially affecting mobility. Thus the study finally included 94 VLBW subjects and 101 controls.

### Non-participant analysis

The non-participant analyses were performed separately for the VLBW and control groups. We first compared the adult characteristics of participants included in the data analysis of this study (94 VLBW and 101 controls) with those 72 VLBW and 71 control subjects who were invited but chose to not participate or had an exclusion criterion. All these subjects had participated in the first clinical visit and thus had data collected in adult life. No differences were seen in socioeconomic status as indicated by parental education, or in height or BMI (all p-values ≥0.1).

Furthermore we compared perinatal characteristics between the 94 VLBW and 101 control participants of the present study, with the remaining original cohort for whom these data were available, i.e. those invited to the first clinical examination (161 VLBW and 213 control subjects). There were no differences in gestational age, birth weight, sex, preeclampsia or multiple pregnancy between the participants who were included in the current study and those who were not (all p-values ≥0.1).

### Clinical data

During the clinical visit, the participants completed a detailed physical activity questionnaire – the modified Kuopio Ischemic Heart Disease Risk Factor Study–questionnaire. The reproducibility and validity of the 12-month physical activity history questionnaire has previously been confirmed in the United States [28], Belgium [29] as well as Finland [30], [31]. This questionnaire presents a 30-item list of different physical activity types, including conditioning LTPA (20 items; e.g. running, skiing, swimming), non-conditioning LTPA (8 items; e.g. household work, gardening, shoveling snow), physical activity from commuting to work (walking or cycling) and an additional category for “other” physical activities specified by the participant. The participants reported monthly frequency and duration of each physical activity session covering the previous 12 months. They also reported the average intensity of activity sessions on a scale from 0 to 3 (0 = light, 1 = moderate, 2 = strenuous, 3 = very strenuous).

During the clinical visit weight and height of each participant was measured, and body mass index (BMI) was calculated [weight (kg)/height squared (m^2^)]. The participants also completed a questionnaire enquiring their smoking habits, illnesses and medications. As the study participants were young adults, still studying or in the beginning of their carrier, the highest education of either parent was enquired and used to describe their socioeconomic status.

A subset of participants (91 VLBW and 88 control subjects) had their body composition measured by dual-energy x-ray absorptiometry (DXA, Discovery A, Hologic) during the first clinical visit (2004–2005). At that time personality traits were evaluated with the 180-item NEO-Personality Inventory [32] based on the Big Five taxonomy, the most commonly used classification of personality traits (conscientiousness, openness to experience, agreeableness, extraversion, and neuroticism) on a 5-point scale (0 = very untrue; 4 = very true). These data were available for 90 VLBW and 98 control subjects.

### Ethics

The study was performed according to the declaration of Helsinki. The study protocol was approved by the Ethics committee at the Helsinki and Uusimaa Hospital District. Written informed consent was obtained from each participant.

### Data Analysis

Self-reported monthly frequency of physical activity was transferred into units of times/year, and duration of each physical activity session summed and transferred into units of minutes/year (total time). Self-rated physical activity intensities were transferred into metabolic equivalents (MET) using standardized activity-specific tables presented in detail elsewhere [33], [34]. By definition a MET is the ratio of metabolic rate during exercise to metabolic rate at rest. 1 MET corresponds to an EE of approximately 1 kcal/kg/hour, this being roughly equivalent to the energy cost of sitting quietly. MET values were used to calculate total volume of physical activity (METh/year) as follows: MET×hours of physical activity/year. Yearly EE (kcal/year) was calculated as total time of physical activity (min/year)×MET (kcal/kg/min/year)×weight (kg).

Based on MET values, we additionally categorized physical activity into vigorous physical activity, including conditioning LTPA, non-conditioning LTPA and commuting activity with MET ≥5.

### Statistical methods

Statistical tests were carried out using PASW Statistics 17 (Chicago IL, USA). Descriptive data for predictor variables and covariates are reported as n (%) or mean (SD). The outcome variables (physical activity) were log-transformed [^10^log(variable+1)] to attain normality. Accordingly, descriptive data for outcome variables are reported as geometric means (geometric mean of n+1 subtracted by 1; the geometric mean denotes the n^th^ root of the product of n individual values) and SDs (geometric standard deviation of n+1; the geometric standard deviation denotes the relative increase in a variable corresponding to one standard deviation unit change in the logarithm of the variable). This is done in average units of physical activity per week to make the numbers easier to interpret.

Baseline characteristics between VLBW and control subjects were compared using t-test for continuous and χ^2^-test for categorical variables. Linear regression was used to compare differences in yearly frequency, total time, total volume and EE of each physical activity subtype (conditioning, non-conditioning, commuting and vigorous physical activity) between VLBW and control subjects. We adjusted for age and sex in model 1; for age, sex and BMI in model 2; and for age, sex, BMI, daily smoking and highest education of either parent in model 3. We additionally adjusted for lean body mass instead of BMI in the subgroup with available data on DXA. Further, conditioning LTPA between VLBW and term was compared by adjusting for model 3 covariates and the mean scores of the five personality trait ratings (model 4). Median imputation was carried out for highest parental education and smoking status which were missing for one control subject. The results are presented as mean differences (%) and 95% confidence intervals (CI) between VLBW subjects and controls.

Furthermore, the influence of each personality trait on physical activity was analyzed by incorporating the score of each trait one-at-a-time in model 1 (adjustment for age and sex) comparing the frequency, total time, total volume and EE of each physical activity subtype between VLBW and term-born subjects. These results are presented as correlation coefficients. To assess whether the influence of personality traits on physical activity was different among VLBW subjects as compared with controls we examined interactions between the effects of VLBW status and personality traits on conditioning LTPA by including a product term (VLBW*personality) in the regression model (model 1).

## Results

Characteristics of study participants are presented in Table 1. Gestational age at birth of VLBW subjects ranged between 24.7 and 35.6 (mean 29.5) weeks and of term-born controls between 37.0 and 42.3 (mean 40.1) weeks. Birth weights ranged between 600 and 1480 (mean 1157) g and between 2560 and 4930 (mean 3608) g, respectively. As young adults, VLBW subjects were shorter than controls. In the subgroup with available data, lean body mass was lower in both men and women born at VLBW than in controls. Daily smoking was less common in VLBW subjects compared with controls (16% vs. 31%, p = 0.02).

**Table 1:** Characteristics of the study participants.

Characteristic	VLBW	Term	*P* a	Missing values,
	(n = 94)	(*n* = 101)		VLBW/Term
**Birth**				
Gestational age, mean (SD), week	29.5 (2.3)	40.1 (1.1)	**<.001**	0/0
Birth weight, mean (SD), g	1157 (208.7)	3608 (492.0)	**<.001**	0/0
Birth weight SDS, mean (SD)	−1.3 (1.5)	0.1 (1.1)	**<.001**	0/0
Women, n (%)	57 (60.6)	59 (58.4)	.8	0/0
Men, n (%)	37 (39.4)	42 (41.6)	.8	0/0
SGA^b^, n (%)	35 (37.2)	0		0/0
Preeclampsia, n (%)	24 (25.5)	9 (8.9)	**.002**	0/0
Twin, n (%)	14 (14.9)	0		0/0
Triplet, n (%)	2 (2.1)	0		0/0
**Current**				
Age, mean (SD), y	24.9 (2.1)	25.1 (2.2)	.6	0/0
Height, mean (SD), cm				
Women	163.0 (7.4)	166.4 (6.2)	**.009**	0/0
Men	176.2 (7.2)	180.8 (6.1)	**.003**	0/0
Body mass index, mean (SD), kg/m^2^				
Women	21.8 (3.7)	22.9 (4.3)	.1	0/0
Men	22.4 (3.5)	23.0 (2.9)	.4	0/0
Lean mass, mean (SD), kg				
Women	39.5 (5.5)	42.8 (6.3)	**.004**	1/5
Men	55.0 (6.7)	61.5 (8.0)	**.001**	2/8
Daily smoking, n (%)	15 (16.0)	31 (30.7)	**.02**	0/1
Parental education^c^, n (%)			.5	0/1
Elementary	8 (8.5)	5 (5.0)		
High school	21 (22.3)	19 (18.8)		
Intermediate	32 (34.0)	33 (32.7)		
University	33 (35.1)	44 (43.6)		

VLBW = very low birth weight (<1500 g).

a The t-test for continuous and chi-square test for categorical variables.

b SGA, small for gestational age, birth weight <−2 SD.

c Highest current education of either parent.

### Physical activity in VLBW and control subjects

Table 2 shows the mean frequency, total time, total volume and EE of each physical activity subtype, as well as the mean frequency, total time and EE of vigorous physical activity in VLBW and term-born groups.

**Table 2:** Weekly frequency, total time, total volume and energy expenditure of different physical activity subtypes and vigorous physical activity in unimpaired preterm-born VLBW and term-born control young adults.

Type of physical activity		VLBW	Term
		(*n* = 94)	(*n* = 101)
		Mean^a^ (SD^b^)	
**Conditioning leisure-time physical activity**	Frequency, times/week	1.4 (0.8)	2.1 (1.0)
	Total time, minutes/week	48.4 (3.9)	84.9 (2.9)
	Total volume, METh/week^c^	7.2 (2.0)	11.4 (1.7)
	Energy expenditure, kcal/week	257.8 (7.2)	585.0 (3.9)
**Non-conditioning leisure-time physical activity**	Frequency, times/week	1.3 (0.9)	1.3 (0.7)
	Total time, minutes/week	55.7 (3.9)	62.6 (2.7)
	Total volume, METh/week^c^	3.8 (1.5)	4.0 (1.1)
	Energy expenditure, kcal/week	173.9 (5.6)	227.2 (3.7)
**Commuting physical activity**	Frequency, times/week	1.8 (1.5)	1.8 (1.6)
	Total time, minutes/week	15.0 (6.7)	14.6 (7.2)
	Total volume, METh/week^c^	5.1 (3.2)	5.2 (3.4)
	Energy expenditure, kcal/week	44.0 (13.0)	45.0 (15.2)
**Vigorous physical activity** d	Frequency, times/week	1.5 (1.0)	1.9 (0.9)
	Total time, minutes/week	0,1 (0.0)	0,1 (0.0)
	Total volume, METh/week^c^	0.1 (0.0)	0.1 (0.0)
	Energy expenditure, kcal/week	240.5 (8.3)	529.9 (4.6)

Physical activity is expressed in units/week in this table to make the numbers easier to interpret.

VLBW = very low birth weight (<1500 g).

a geometric mean of n+1 subtracted by 1;geometric mean denotes the n^th^ root of the product of n individual values.

b geometric standard deviation of n+1; geometric standard deviation denotes the relative increase in a variable corresponding to one standard deviation unit change in the logarithm of the variable.

c MET×hours of physical activity/week (MET = metabolic equivalents; ratio of metabolic rate during exercise and estimated metabolic rate at rest; 1 MET corresponds energy expenditure of approximately 1 kcal/kg×hour).

d all physical activity with MET ≥5, including conditioning, non-conditioning and commuting activity.

Differences between VLBW and term are presented in Table 3.

### Conditioning leisure-time physical activity

The frequency, total time, total volume and EE of conditioning LTPA was significantly lower in VLBW compared with control subjects (Table 3). Adjustment for BMI, smoking and highest parental education had little effect on this difference. When we adjusted for lean body mass instead of BMI in the subgroup of subjects who underwent DXA scans, differences in the frequency [−17.4% (95% CI; −45.7, 25.9), p = 0.37], total time [−26.2% (95% CI; −60.6, 38.4) p = 0.34], total volume [−23.1% (95% CI; −53.7, 27.4) p = 0.31] and EE [−33.0% (95% CI; −68.6, 42.6) p = 0.30] of conditioning LTPA were no longer statistically significant.

**Table 3:** Differences in frequency, total time, total volume and energy expenditure of different physical activity subtypes between unimpaired preterm-born VLBW and term-born control young adults.

Mean difference (%) and 95% confidence interval between VLBW (*n* = 94) and term-born (*n* = 101) subjects					
		Frequency	Total time	Total volume	Energy expenditure
		(times/year)	(minutes/year)	(METh/year^a^)	(kcal/year)
**Conditioning leisure-time physical activity**	Model 1	−41.8 (−60.9, −13.1)^c^	−51.2 (−73.1, −11.5)^c^	−48.6 (−68.4, −16.2)^c^	−63.0 (−82.1, −23.4)^c^
	Model 2	−40.0 (−59.9, −10.3)^c^	−48.8 (−71.9, −6.9)^c^	−46.0 (−66.9, −12.1)^c^	−58.9 (−80.0, −15.1)^c^
	Model 3	−38.5 (−58.9, −7.7)^c^	−47.4 (−71.2, −4.1)^c^	−44.3 (−65.8, −9.2)^c^	−55.9 (−78.6, −9.4)^c^
**Non-conditioning leisure-time physical activity**	Model 1	−14.7 (−41.0, 23.6)	−21.8 (−57.9, 45.2)	−20.0 (−48.1, 23.6)	−32.2 (−66.4, 36.8)
	Model 2	−13.5 (−40.4, 25.6)	−20.6 (−57.4, 48.6)	−18.5 (−47.9, 26.5)	−28.2 (−64.6, 45.2)
	Model 3	−4.5 (−34.5, 39.6)	−4.3 (−49.2, 79.9)	−8.4(−41.3, 43.2)	−12.1 (−57.0, 79.9)
**Commuting physical activity**	Model 1	0.4 (−50.3, 103.7)	8.1 (−62.2, 209.7)	1.2 (−57.6, 142.1)	3.3 (−70.2, 258.1)
	Model 2	2.1 (−50.0, 108.0)	11.4 (−61.5, 222.1)	3.3 (−57.1, 148.9)	9.9 (−68.7, 285.5)
	Model 3	6.2 (−48.7, 119.8)	16.9 (−60.5, 246.7)	6.7 (−56.6, 162.4)	14.3 (−68.2, 314.0)
**Vigorous physical activity** b	Model 1	−36.8 (−59.0, −2.5)^c^	−11.5 (−23.4, 2.3)	−11.5 (−23.4, 2.6)	−63.5 (−83.6, −18.5)^c^
	Model 2	−35.6 (−58.3, −0.2)^c^	−10.1 (−22.4, 4.0)	−10.3 (−22.6, 4.0)	−60.0 (−82.1, −10.7)^c^
	Model 3	−34.7 (−58.0, 1.9)	−8.8 (−21.3, 5.7)	−9.2 (−21.7, 5.4)	−55.3 (−80.1,0.5)

VLBW = very low birth weight (<1500 g).

a MET×hours of physical activity/year (MET = metabolic equivalents; ratio of metabolic rate during exercise and estimated metabolic rate at rest; 1 MET corresponds energy expenditure of approximately 1 kcal/kg×hour).

b physical activity with MET ≥5.

c *P*<.05.

Model 1; adjusted for age and sex.

Model 2; adjusted for age, sex and body mass index.

Model 3; adjusted for age, sex, body mass index, daily smoking of the participant, and highest education of either parent.

Predicted by linear regression and adjusted for covariates in different models.

Adjustment for the five personality traits in model 4 slightly increased the difference in all dimensions (frequency, total time, total volume and EE) of conditioning LTPA between VLBW and control subjects (Table 4).

**Table 4:** Differences in frequency, total time, total volume and energy expenditure of conditioning leisure-time physical activity between unimpaired preterm-born VLBW (n = 90) and term-born control (n = 98) young adults obtained by linear regression.

	Frequency (times/year)	Total time (minutes/year)	Total volume (METh/year^a^)	Energy expenditure (kcal/year)
**Model 4: difference between VLBW and term**	**−48.1 (−64.8, −23.6)** c	**−60.5 (−77.7, −30.2)** c	**−54.9 (−71.6, −28.4)** c	**−68.4 (−84.0, −37.2)** c
	**Unstandardized regression coefficient (95% confidence interval)**			
Age	2.3 (−6.7, 11.9)	5.9 (−7.3, 21.1)	3.8 (−7.1, 15.6)	8.4 (−7.5, 27.4)
Sex^b^	−42.3 (−63.9, −7.7)^c^	−55.8 (−77.9, −11.9)^c^	−47.5 (−70.1, −8.0)^c^	−55.7 (−80.8, 1.9)
BMI	2.8 (−2.2, 8.1)	7.2 (−2.9, 13.0)	5.0 (−1.4, 11.4)	12.5 (2.6, 23.0)^c^
Daily smoking	−13.5 (−46.7, 40.6)	−22.2 (−61.9, 58.9)	−15.1 (−52.4, 51.7)	−15.9 (−64.4, 98.6)
Parental education^d^	31.8 (6.2, 64.1)^c^	52.8 (10.9, 110.4)^c^	41.3 (8.9, 83.2)^c^	86.6 (27.1, 174.2)^c^
Extraversion	57.8 (−1.8, 153.3)	111.8 (5.2, 325.6)^c^	73.0 (−2.1, 204.8)	118.8 (−5.8, 407.0)
Openness to experience	−27.7 (−54.2, 15.6)	−48.2 (−73.8, 2.3)	−34.8 (−62.5, 13.5)	−53.9 (−79.7, 4.7)
Neuroticism	−27.7 (−52.7, 10.2)	−34.5 (−64.9, 21.9)	−31.8 (−58.9, 13.0)	−45.7 (−74.4, 14.8)
Agreeableness	68.3 (3.8, 173.5)^c^	145.5 (19.9, 402.3)^c^	112.8 (19.1, 280.2)^c^	174.2 (15.9, 547.1)^c^
Conscientiousness	−1.8 (−31.0, 39.6)	−12.5 (−47.9, 47.2)	−9.4 (−40.6, 38.0)	−16.2 (−55.1, 56.7)

VLBW = very low birth weight (<1500 g).

Model 4: adjusted for age, sex, body mass index, daily smoking, highest education of either parent, extraversion, openness to experience, neuroticism, agreeableness, and conscientiousness (for Models 1, 2 and 3, see Table 3).

a MET×hours of physical activity/year (MET = metabolic equivalents; ratio of metabolic rate during exercise and estimated metabolic rate at rest; 1 MET corresponds energy expenditure of approximately 1 kcal/kg×hour).

b Women = 0; Men = 1.

c *P*<.05.

d Highest education of either parent; Elementary = 1; High school = 2; Intermediate = 3; University = 4.

Adjusted for covariates of Table 3 plus personality characteristics, with the association of each covariate shown.

Of the covariates, sex had a significant influence on frequency, total time and total volume of conditioning LTPA, with lower values in men than in women (Table 4). BMI had a significant positive association with EE, and highest parental education on all dimensions of conditioning LTPA. The associations with personality traits are presented below.

### Non-conditioning leisure-time and commuting physical activity

Yearly frequency, total time, total volume or EE of non-conditioning LTPA or commuting physical activity did not differ significantly between VLBW and term-born subjects in models 1 through 3 (Table 3). Adjustment for lean body mass in the subgroup with data available did not change these results (data not shown).

### Vigorous physical activity

Yearly frequency and EE of high intensity (MET ≥5) physical activity were significantly lower in VLBW than in control subjects (Table 3) when adjusting for age, sex and BMI (models 1 and 2), but not after further adjustments for smoking and parental education (model 3). No significant differences were seen in the total time or volume of vigorous physical activity.

To estimate the effect of fetal growth on physical activity, we also compared all physical activity data between those VLBW subjects who were born small for gestational age (SGA, birth weight <−2SD, *n* = 35) and those VLBW subjects who were born appropriate for gestational age (AGA, birth weight ≥−2SD, *n* = 59) [35]. No significant differences were revealed (data not shown).

There was no difference in physical activity between the 19 VLBW subjects with a history of bronchopulmonary dysplasia and the 75 VLBW subjects without bronchopulmonary dysplasia.

When we re-analyzed the data after further exclusion of 16 VLBW adults and 6 control subjects with a history of asthma the results remained similar.

### Physical activity and personality

Table 5 shows correlation coefficients representing the impact of the five personality traits on physical activity subtypes obtained by incorporating personality scores one-at-a-time in linear regression (model 1). Extraversion and agreeableness had a significant positive, and neuroticism a negative correlation with all dimensions of conditioning LTPA. Extraversion had a negative, and openness to experience a positive correlation with commuting physical activity. No significant correlations were found between any personality trait and non-conditioning LTPA. Of the personality traits, conscientiousness was not correlated with any physical activity subtype.

**Table 5:** Correlation coefficients (standardized regression coefficients) representing the impact of the five personality traits on physical activity, obtained by linear regression comparing VLBW and control subjects (adjusted for sex and age).

	Frequency (times/year)	Total time (minutes/year)	Total volume (METh/year^a^)	Energy expenditure (kcal/year)
	**Conditioning leisure-time physical activity**			
Extraversion	0.24^c^	0.23^c^	0.23^c^	0.23^c^
Conscientiousness	0.09	0.07	0.07	0.05
Neuroticism	−0.28^d^	−0.27^c^	−0.28^d^	−0.26^c^
Agreeableness	0.21^c^	0.22^c^	0.23^c^	0.19^c^
Openness to experience	0.03	0.00	0.03	0.00
	**Non-conditioning leisure-time physical activity**			
Extraversion	0.11	0.12	0.10	0.12
Conscientiousness	0.07	0.03	0.04	0.02
Neuroticism	−0.08	−0.07	−0.05	−0.05
Agreeableness	0.08	0.08	0.08	0.07
Openness to experience	0.04	0.04	0.04	0.04
	**Commuting physical activity**			
Extraversion	−0.15^b^	−0.16^b^	−0.17^b^	−0.17^b^
Conscientiousness	0.11	0.10	0.10	0.09
Neuroticism	−0.07	−0.06	−0.06	−0.06
Agreeableness	0.14	0.14	0.14	0.13
Openness to experience	0.23^c^	0.23^c^	0.23^c^	0.23^c^

Correlation coefficient is equal to a standardized regression coefficient: a 1 standard deviation unit higher score on a personality trait is associated with the correlation coefficient×standard deviation unit physical activity.

VLBW = very low birth weight (<1500 g).

a MET×hours of physical activity/year (MET = metabolic equivalents; ratio of metabolic rate during exercise and estimated metabolic rate at rest; 1 MET corresponds energy expenditure of approximately 1 kcal/kg×hour).

b P<.05.

c P<.01.

d P<.001.

**Figure 1:**
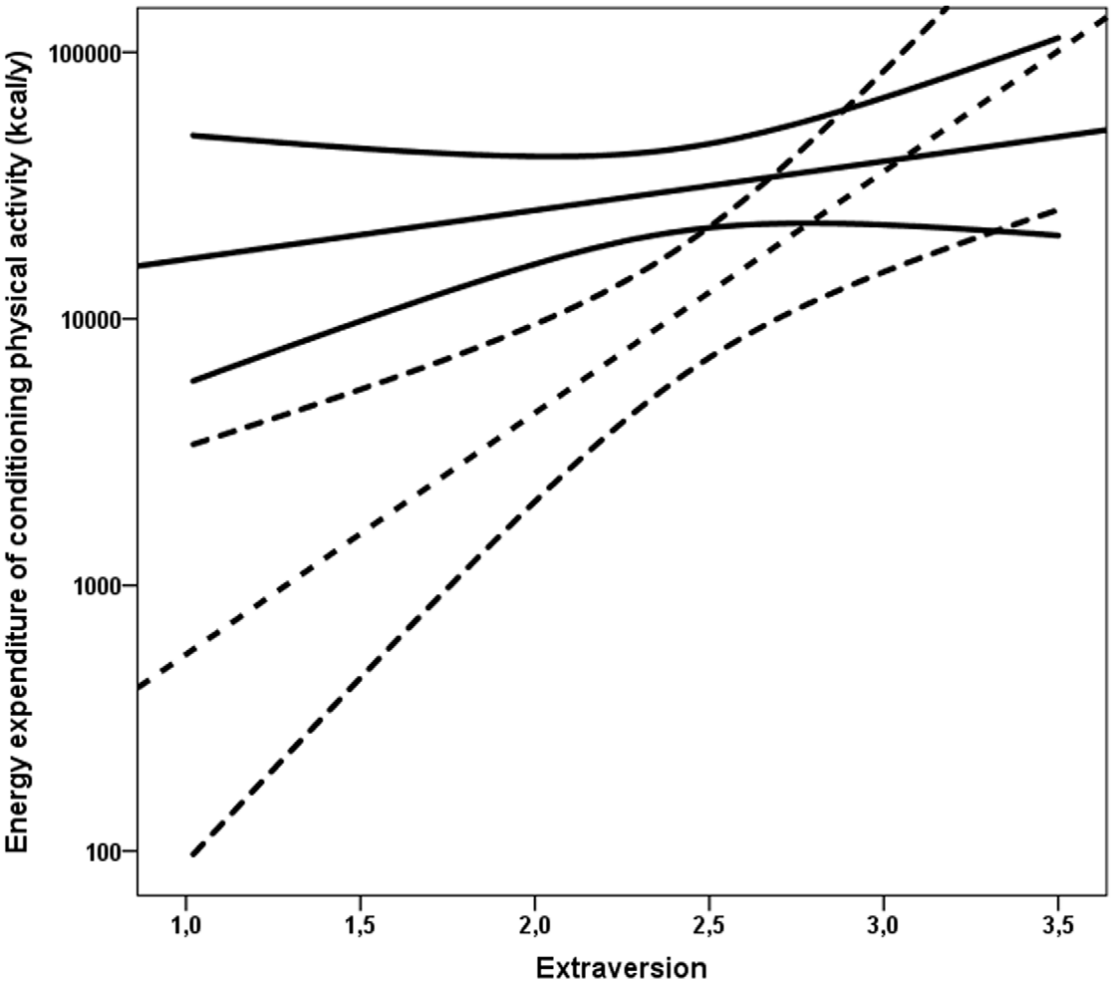
The interactions between the effects of extraversion and birth status on yearly energy expenditure. The association between extraversion and yearly energy expenditure of conditioning leisure-time physical activity among subjects born at very low birth weight (<1500 g) (dashed lines) and at term (continuous lines). The lines denote regression slopes and 95% confidence intervals. The higher end of the scale represents the most extraverted individuals. The effects of extraversion and VLBW status on energy expenditure in conditioning leisure-time conditioning physical activity are different between VLBW and term born subjects.

We observed statistically significant interactions between the effects of extraversion and VLBW status on total volume (p for interaction = 0.02) and EE (p for interaction = 0.02) of conditioning LTPA (Figure 1). No other interactions between the effects of VLBW birth and personality on conditioning LTPA were seen (p for interaction >0.05). Investigating the impact of extraversion on EE of conditioning LTPA separately in VLBW and control subjects revealed that the relationship between extraversion and EE of conditioning LTPA was stronger among VLBW subjects than among controls: correlation coefficients (standardized regression coefficients) were 0.34 among the VLBW and 0.11 among controls.

## Discussion

We found that unimpaired young adults born at VLBW participate markedly less in conditioning LTPA than their peers born at term. Of the components of conditioning LTPA, the frequency, total time, total volume and EE were all lower in VLBW subjects than in term-born controls. There were no differences in non-conditioning LTPA or in commuting physical activity. The results were not affected by age, sex, BMI, daily smoking, socioeconomic status or personality traits.

A number of previous studies have assessed the association between gestational age at birth or birth weight and later physical activity. As to birth weight, a large meta-analysis of adolescents and adults, including subjects with birth weights ranging from 1.26 kg to 5.25 kg, showed that people at both ends of the birth weight spectrum were less physically active [36]. The lowest end of the birth weight distribution is likely to represent people born preterm. Other studies have assessed physical activity specifically in people born as small preterms. Rogers et al. (2005) investigated unimpaired adolescents born preterm at extremely low birth weight (≤800 g) [22]. They reported that, in comparison with controls, these subjects participated less in physical activities and had lower muscle strength and flexibility, probably relating to immaturity of the motor system. Also in extremely low birth weight (≤1000 g) survivors in their young adulthood, Saigal et al. (2007) reported lower scores on physical efficacy, self-perceived physical ability and physical self-confidence than in controls born at term [21]. Lower levels of sports participation have also been reported in subjects born at VLBW [10], [20].

From an earlier examination of the same VLBW-cohort we now studied, Kajantie et al. (2010) reported lower participation in LTPA compared with controls [23]. However, in that study the evaluation of physical activity was based only on 9 general questions in contrast to the present study in which a much more detailed questionnaire was used. In the present study we took into consideration the multidimensionality of physical activity in more detail as we separately studied yearly frequency, total time, total volume and EE of each physical activity subtype during the previous 12 months. In conditioning LTPA all these were lower in VLBW than in control subjects, although there were no differences in non-conditioning LTPA or in commuting physical activity. Furthermore, we separately looked at high intensity physical activity. VLBW subjects tended to participate less often in vigorous physical activity although statistical significance was not attained after adjustment for all covariates. In addition, we evaluated the effects of personality on physical activity and found that adjustment for personality traits did not change the results. The lower participation in conditioning LTPA offers a potential mechanism linking preterm birth at VLBW and increased risk factors for chronic adult disease and must be taken into account in future designing of preventive measures.

VLBW subjects have lower lean body mass [1] than their term-born peers. This seemed in part to underlie the difference in conditioning LTPA in our study, as the difference was attenuated after adjustment for lean body mass. However, it is difficult to distinguish between cause and consequence. In addition to lower lean body mass, subjects born severely preterm have lower muscle strength [22], [37], exercise capacity [22], [38], [39], poorer motor coordination [22], [39], [40] and visual acuity [40], all present from childhood onwards. These characteristics are likely to make physical activity less rewarding, resulting in physical inactivity, which aggravates the slower development of motor skills and contributes to the lower muscle and lean body mass.

VLBW subjects' lesser participation in physical activity could also potentially be explained by differences in personality. However, while some of the characteristic personalities of VLBW adults (inhibition, risk avoidance) [24], [41] are expected to be associated with reduced levels, others (conscientiousness) are generally associated with higher levels of physical activity [25]. We did not find an association between conscientiousness and physical activity, but in accordance with previous literature [25], extraversion had a significant positive, and neuroticism a negative association with conditioning LTPA. Conditioning LTPA was also associated with higher agreeableness. Incorporating personality traits as covariates in the linear regression did not dilute the difference in conditioning LTPA between VLBW subjects and controls; on the contrary the difference increased. Thus, personality differences between VLBW and control subjects, as captured by the Big Five taxonomy, do not explain our results of lower conditioning LTPA in VLBW subjects.

It is of note that the widely replicated relationship between extraversion and physical activity [25] was stronger among VLBW subjects than among controls born at term. As a result of this, the difference in physical activity between VLBW and term was strongest among the most introverted subjects. While we have no clear explanation for this finding, it may be important when planning physical activity interventions in subjects born at VLBW.

Our results were not explained by age, sex, BMI, daily smoking or socioeconomic status.

Prenatal environment of VLBW subjects born SGA is frequently characterized by intrauterine growth restriction and is thus a lot different from that of those born AGA while postnatal challenges are to a great extent similar. Therefore, the finding that the lower conditioning LTPA was found similarly in both SGA and AGA VLBW subjects suggests that the differences in physical activity are a result of postnatal events or prematurity itself rather than prenatal conditions.

### Limitations

One key limitation of our study is the relatively small sample size which is, however, comparable to or larger than in may previous studies with related outcomes [10], [22], [39]. Moreover, the study participants may not be representative of the original cohort, although non-participant analyses showed no differences in perinatal characteristics, height, BMI or socio-economic status. Furthermore, this would only be expected to introduce bias if the association between VLBW and physical activity was different among participants than among non-participants. This is unlikely, but cannot be excluded.

Information of physical activity was self-reported instead of using objective assessment, potentially introducing inaccuracy. However, all questionnaires rely on the validity of self-report, all types of physical activity cannot be measured by objective measurement and potential inaccuracy of self-reported data should in part be overcome by the great detail of the physical activity questionnaire. Moreover, VLBW subjects tend to adjust their responses to produce socially more favorable answers which might affect the results [42]. Cardiorespiratory fitness of the participants was not assessed, lower cardiorespiratory fitness would be expected with lower levels of physical activity. As the treatment of prematurely born neonates today differs from the late 1970s and 1980s, when the subjects of our cohort were born, the results may not be directly applied to the present. Current treatment has more to offer, which hopefully leads to healthier next generation VLBW subjects. As the aim was to investigate physical activity in unimpaired VLBW subjects, the full extent of physical inactivity in all individuals born at VLBW might be underestimated.

### Conclusions

Adults who were born preterm at VLBW undertake less LTPA than their peers born at term. Differences in personality characteristics that exist between VLBW and term-born groups do not explain the lower levels of physical activity. Since preterm birth at VLBW is associated with increased risk factors of type 2 diabetes [1], [2], hypertension [4]–[7], osteoporosis [9] as well as impaired lung function in adulthood [10], conditions that all may be prevented by being physically fit [15]–[18], it is important to motivate people born prematurely at VLBW to a physically active lifestyle.
